# Effects of Inhaled Brevetoxins in Allergic Airways: Toxin–Allergen Interactions and Pharmacologic Intervention

**DOI:** 10.1289/ehp.7498

**Published:** 2005-02-10

**Authors:** William M. Abraham, Andrea J. Bourdelais, Ashfaq Ahmed, Irakli Serebriakov, Daniel G. Baden

**Affiliations:** ^1^Division of Pulmonary and Critical Care Medicine, University of Miami at Mount Sinai Medical Center, Miami Beach, Florida, USA;; ^2^Center for Marine Science, University of North Carolina at Wilmington, Wilmington, North Carolina, USA

**Keywords:** animal models, asthma, brevetoxin, bronchoconstriction, clinical therapies

## Abstract

During a Florida red tide, brevetoxins produced by the dinoflagellate *Karenia brevis* become aerosolized and cause airway symptoms in humans, especially in those with pre-existing airway disease (e.g., asthma). To understand these toxin-induced airway effects, we used sheep with airway hypersensitivity to *Ascaris suum* antigen as a surrogate for asthmatic patients and studied changes in pulmonary airflow resistance (*R*_L_) after inhalation challenge with lysed cultures of *K. brevis* (crude brevetoxins). Studies were done without and with clinically available drugs to determine which might prevent/reverse these effects. Crude brevetoxins (20 breaths at 100 pg/mL; *n* = 5) increased *R*_L_ 128 ± 6% (mean ± SE) over baseline. This bronchoconstriction was significantly reduced (% inhibition) after pretreatment with the glucocorticosteroid budesonide (49%), the β _2_ adrenergic agent albuterol (71%), the anticholinergic agent atropine (58%), and the histamine H_1_-antagonist diphenhydramine (47%). The protection afforded by atropine and diphenhydramine suggests that both cholinergic (vagal) and H_1_-mediated pathways contribute to the bronchoconstriction. The response to cutaneous toxin injection was also histamine mediated. Thus, the airway and skin data support the hypothesis that toxin activates mast cells *in vivo*. Albuterol given immediately after toxin challenge rapidly reversed the bronchoconstriction. Toxin inhalation increased airway kinins, and the response to inhaled toxin was enhanced after allergen challenge. Both factors could contribute to the increased sensitivity of asthmatic patients to toxin exposure. We conclude that *K. brevis* aerosols are potent airway constrictors. Clinically available drugs may be used to prevent or provide therapeutic relief for affected individuals.

Florida red tide is a harmful algal bloom caused by the dinoflagellate *Karenia brevis* (previously *Gymnodinium breve*). *K. brevis* produces at least nine structurally related poly-ether brevetoxins (PbTxs; Baden 1989; Baden et al. 1995; Pierce and Kirkpatrick 2001), which are lipid-soluble, fused polyethers with molecular weights of approximately 900 Da. During red tide events, toxins released from disrupted organisms are concentrated in seawater droplets that subsequently can become aerosolized (Pierce et al. 1990, 2003). Onshore winds then carry these aerosols inland, where exposed individuals report both upper and lower airway symptoms, such as nonproductive cough, shortness of breath, rhinorrhea, and sneezing (Asai et al. 1982; Backer et al. 2003; Kirkpatrick et al. 2004). There is a suggestion that the frequency of these adverse respiratory events is increased in “susceptible populations,” that is, in those with pre-existing airway disease, as indicated from a clinical survey where 80% of patients with bronchial asthma were reportedly affected during a red tide event, with some having overt asthma attacks (Asai et al. 1982).

Although the data from these clinical surveys and our own field studies (Backer et al. 2003; Baden and Tomas 1988; Fleming et al. 2005; Kirkpatrick et al. 2004; Pierce et al. 1992, 2003) indicate that aerosolized toxins are respiratory irritants and that the effects may be more severe in asthmatics, there is a paucity of data examining the effects of aerosolized toxin in asthmatic airways under controlled conditions. To address this problem, we initially studied the airway responses to inhaled PbTxs in a sheep model of asthma (Abraham et al. 2005). This model shares many characteristics of the disease in humans, including the development of early airway responses (EAR) and late airway responses (LAR) and postantigen-induced airway hyper-responsiveness (AHR) after inhalation challenge with *Ascaris suum* antigen (Abraham 2000). The model also demonstrates nonspecific bronchial hyperresponsiveness to a variety of agents, and we have previously used it to study the pulmonary consequences of pollutant exposures (Abraham et al. 1980, 1981). Furthermore, the antigen-induced effects in this model can be ameliorated with the current armamentarium of clinically available asthma medications, including glucocorticosteroids, β _2_ adrenergic agents, and leukotriene antagonists (Abraham 2000). Collectively, the characteristics of the model suggest that it can be used as a surrogate for patients with compromised airways to study the effects of inhaled toxin.

In our initial studies, allergic sheep that inhaled environmentally relevant concentrations (picogram per milliliter) of lysed cultures of *K. brevis* (i.e., crude brevetoxins, which contains all toxins and cell debris) or purified PbTx-2 or PbTx-3 developed significant bronchoconstriction (Abraham et al. 2005). The magnitude of the response was similar for the three toxins (Abraham et al. 2005). Previous *in vitro* studies using canine tracheal and human bronchial smooth muscle (Asai et al. 1982; Shimoda et al. 1988), demonstrated that brevetoxin-induced contractile effects can be blocked with atropine but not with a histamine antagonist, suggesting that toxin-induced constriction results from stimulation of parasympathetic postganglionic neurons. In contrast to the prior *in vitro* findings, however, our *in vivo* studies showed that protective effects were achieved with the mast cell stabilizer cromolyn sodium and the histamine H_1_-antagonist diphenhydramine. These data support a role for a histamine H_1_-mediated pathway contributing to the bronchoconstrictor response *in vivo* (Abraham et al. 2005). Because mast cells and basophils are the most prominent source of histamine in the airways, our findings suggest that toxin, either directly or indirectly, causes mast cell/basophil activation. Such a mechanism could in part explain the reported increased incidence of asthma attacks after natural red tide episodes.

Another factor that could play a role in asthma exacerbations is the generation of kinins in the airway. The pattern of airway responses and the pharmacologic profile seen with inhaled toxin (Abraham et al. 2005) are similar to those seen previously by us with inhaled bradykinin (Abraham et al. 1991). Increased airway kinin levels occur after exposure to a variety of noxious stimuli (Lauredo et al. 2003), including allergen, ozone, bacterial products, and metabisulfite (Forteza et al. 1994, 1999; Mansour et al. 1992), and are associated with bronchoconstriction, AHR, and lung neutrophilia; all of these responses are seen after inhalation challenge with PbTx-3 (Zaias et al. 2004).

The evidence suggesting that toxin stimulates mast cells/basophils and/or increases airway kinins heightens the importance of understanding toxin effects in allergic airways. Therefore, in this study we used allergic sheep to study further the effects of inhaled toxin in compromised airways. Specifically, we determined *a*) if toxin alone could induce airway responses similar to that seen with allergen, *b*) if preexposure to toxin could exacerbate allergen-induced responses, and *c*) the effects of clinically available drugs and experimental pharmacologic agents on toxin-induced airway constriction. In addition, we showed that toxin activates skin mast cells and so provide further support for the *in vivo* mast cell hypothesis.

## Materials and Methods

Adult ewes (Quin Tindall, Okeechobee, FL) that were naturally sensitive to *A. suum* antigen and had demonstrated airway hypersensitivity to this antigen were used (Abraham 2000). The animals were conscious and restrained in a cart in an upright position with their heads immobilized for the described studies. Instrumentation was performed under local anesthesia. The study was conducted at Mount Sinai Medical Center under the approval of the Mount Sinai Medical Center Animal Research Committee.

### Pulmonary resistance.

These methods have been reported in detail (Abraham et al. 1994, 2000, 2004). Briefly, a balloon catheter was advanced through one nostril into the lower esophagus, and the animals were intubated with a cuffed endotracheal tube through the other nostril. Animals remained intubated throughout the course of a particular experiment, but to avoid discomfort during these studies, the cuff of the endotracheal tube was inflated only during the measurements of pulmonary resistance (*R*_L_) and during delivery of nebulized agents. We have shown previously that this intubation procedure does not affect *R*_L_ or airway responsiveness in these animals for periods of up to 9 hr (Russi et al. 1984). Pleural pressure was measured via the esophageal catheter. Lateral pressure in the trachea was measured with a side-hole catheter advanced through and positioned distal to the tip of the endotracheal tube. Transpulmonary pressure, the difference between tracheal and pleural pressure, was measured with a differential pressure transducer. To measure *R*_L_, the proximal end of the endotracheal tube was connected to a pneumotachograph, and the signals of flow and transpulmonary pressure were recorded on a computer. Respiratory volume was obtained by digital integration of the flow signal so that *R*_L_ was calculated from the transpulmonary pressure and flow, at isovolumetric points. Analysis of 5–10 breaths was used for each determination of *R*_L_.

### Agents.

Crude brevetoxins and purified PbTx-3 were obtained from the Center for Marine Science at the University of North Carolina at Wilmington. Crude brevetoxin was diluted in NH-15 buffer. PbTx-3 was first diluted in a small volume of Alkamuls (Emulphor EL-620: ethoxylated castor oil and water; Chemtec Chemical Co., Chatsworth, CA), followed by suspension in phosphate-buffered saline (PBS). Atropine sulfate injection (Baxter Health Care, Deerfield, IL) was given at a dose of 0.2 mg/kg, iv,; the histamine H_1_-antagonist diphenhydramine hydrochloride (Elkins-Sinn Inc., Cherry Hill, NJ) was diluted in PBS and given at a dose of 2 mg/kg, iv. Albuterol sulfate inhalation solution (2.5 mg/3 mL; Dey, Napa, CA) was given as an aerosol. *A. suum* extract (Greer Diagnostics, Lenoir, NC) was diluted with PBS to a concentration of 82,000 protein nitrogen units/mL and delivered as an aerosol (20 breaths/min). The following agents were all obtained from Sigma (St. Louis, MO): The bradykinin B_2_ receptor antagonist HOE-140 was diluted in PBS, and given as an aerosol (400 nM/kg); budesonide was first diluted in 1 mL ethanol and then in PBS to give a 1 mg/3 mL solution and was given as an aerosol; carbamylcholine (carbachol) was dissolved in PBS at concentrations of 0.25, 0.50, 1.0, 2.0, and 4.0% wt/vol and delivered as an aerosol. As reported previously in detail (Abraham et al. 1994, 2004; Scuri et al. 2002), we used a dosimeter-piston ventilator system with a Raindrop nebulizer (Nelcor Puritan Bennett, Carlsbad, CA) to deliver the aerosols directly into the endotracheal tube only during inspiration at a tidal volume of 500 mL and a rate of 20 breaths/minute.

### Assessment of nonspecific airway responsiveness.

Airway responsiveness to carbachol was determined from cumulative concentration–response curves as previously described (Abraham et al. 1994, 2004; Scuri et al. 2002). *R*_L_ was measured immediately after inhalation of PBS and within 5 min after each consecutive administration of 10 breaths of increasing concentrations of carbachol (0.25, 0.5, 1.0, 2.0, and 4.0% wt/vol PBS). The provocation test was discontinued when *R*_L_ increased > 400% from the post-PBS value or after the highest carbachol concentration had been administered. The cumulative carbachol concentration (in breath units) that increased *R*_L_ by 400% over the post-PBS value (PC400) was calculated by interpolation from the dose–response curve. One breath unit was defined as one breath of a 1% wt/vol carbachol aerosol solution. A decrease in the PC400 indicates the development of AHR.

### Airway responsiveness to PbTx-3.

Baseline *R*_L_ was measured, and then the sheep were challenged with 20 breaths of increasing concentrations of PbTx-3: 0.1, 0.3, 1, and 10 pg/mL of PbTx-3. *R*_L_ was measured within 5 min after the delivery of each concentration of toxin.

### Airway responses to toxin.

Baseline *R*_L_ was measured, and then the sheep were challenged with 20 breaths of 100 pg/mL of crude brevetoxins. *R*_L_ was measured immediately after challenge and then 15, 30, and 60 min after challenge. Responses to crude brevetoxins alone were compared with the responses obtained after treating the animals with the histamine H_1_-antagonist diphenhydramine, the anticholinergic agent atropine, the glucocorticosteroid budesonide, or the bradykinin B_2_ receptor antagonist HOE-140. All drugs were given 30 min before challenge. Repeat challenges were separated by a minimum of 48 hr. In a separate study, albuterol aerosol was given immediately after inhalation of crude brevetoxins to determine if the drug could reverse the toxin-induced bronchoconstriction. Drug doses chosen for these studies were based on our previous use of these compounds (Abraham 2000).

### Allergen–PbTx-3 interaction.

To determine if multiple exposures to PbTx-3 affected antigen-induced responses, we challenged sheep in the morning with 20 breaths of 100 pg/mL PbTx-3 for 3 consecutive days and then on the fourth day challenged the animals with allergen to determine if the antigen-induced EAR, LAR, and AHR were affected. For these studies a baseline PC400 to carbachol was determined 1–3 days before the start of PbTx-3 exposures. One day after the last PbTx-3 exposure (day 4), baseline *R*_L_ was measured, and then the sheep were challenged with antigen. *R*_L_ was measured immediately after, hourly for 1–6 hr after, and then on the half-hour from 6.5 to 8 hr after challenge. On the next day, the postantigen PC400 was determined. The results were compared with those obtained without PbTx-3 exposure in the same sheep.

To determine if allergen challenge affected the response to PbTx-3, a baseline PC400 to carbachol and a concentration–response curve to PbTx-3 were determined. One to 3 days later, baseline *R*_L_ was measured, and then the sheep were challenged with antigen. *R*_L_ was measured immediately after, hourly for 1–6 hr after, and then on the half-hour from 6.5 to 8 hr after challenge. On the next day, the postantigen response to PbTx-3 was determined. Two to 3 hr later when *R*_L_ had returned to normal, the postantigen PC400 to carbachol was measured. The changes in the postchallenge compared with prechallenge responses to PbTx-3 and PC400 were assessed to determine if they were affected by the allergen provocation.

### Cutaneous responses.

To further investigate the potential for a generalized H_1_-mediated response to toxin, we performed skin tests with crude brevetoxins and pure PbTx-3 to determine if they would induce a wheal response and if the response was histamine mediated (Lucio et al. 1992; Molinari et al. 1995). Immediate cutaneous responses (ICRs) were induced by intradermal injections of 0.05 mL of 5% wt/vol histamine, 100 pg/mL crude brevetoxins, or PbTx-3 solutions using insulin syringes with 28-gauge needles. These studies were repeated, but the sheep were treated with diphenhydramine (2 mg/kg, iv) 2 hr before intradermal challenge or with atropine (0.2 mg/kg, iv) 30 min before challenge.

### Statistical analysis.

Overall effects of airway responses with and without pharmacologic intervention were analyzed with a multifactorial analysis of variance for repeated measures. If the null hypothesis was rejected, then Tukey’s post hoc test was used to determine the statistical significance of differences. In the event only two treatments were compared, a paired *t*-test was used. Analysis of the ICR was determined as described previously by our group (Molinari et al. 1995; Lucio et al. 1992). Wheal sizes were measured 20 and 60 min after injection of active test solutions. The surface area (mm^2^) of the wheal was determined by measuring the largest wheal diameter (*D*_1_) and its perpendicular (*D*_2_) and then calculating the surface area using the equation Π[(*D*_1_ + *D*_2_)/4]^2^. Results were compared by one-way analysis of variance followed by Tukey’s post hoc test if the null hypothesis was rejected. For all analyses, significance was accepted when *p* < 0.05 using a two-tailed analysis. Values in the text and figures are reported as mean ± SE.

## Results

### Airway responses to toxin with and without pharmacologic intervention.

**Figure 1:**
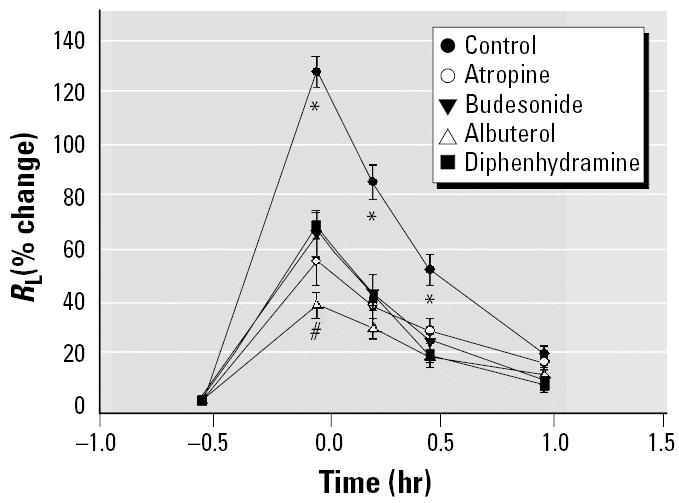
Effect of pharmacologic agents on breve-toxin-induced bronchoconstriction. Crude brevetoxins produced an immediate increase in *R*_L_, which then returned to baseline values within 1 hr. Pretreatment with atropine, budesonide, albuterol, and diphenhydramine all reduced the toxin-induced response. Values are mean ± SE for five sheep. 
*p < 0.05 versus all others; ^#^*p* < 0.05 albuterol versus diphenhydramine.

**Figure 2:**
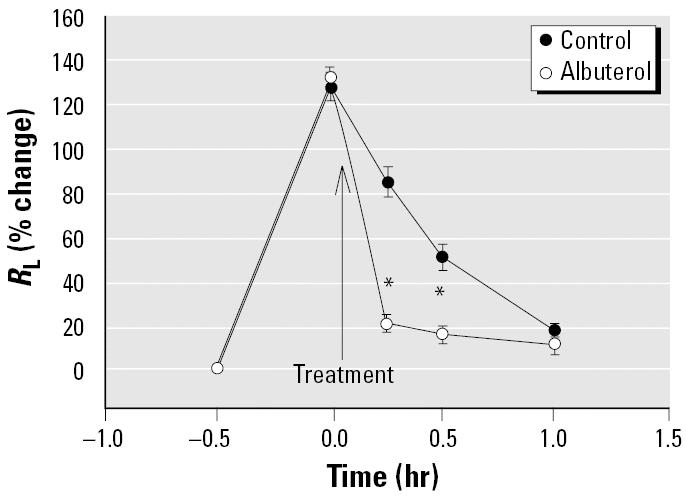
Reversal of crude brevetoxin–induced bronchoconstriction with albuterol. Values are mean ± SE for five sheep. 
*p < 0.05 versus untreated.

Inhalation of crude brevetoxins resulted in an immediate bronchoconstriction (*R*_L_ increased 128 ± 6% over baseline), which then resolved over the next 60 min (Figure 1). The constrictor effects of toxin were significantly reduced by pretreating the animals with the anticholinergic agent atropine (58% inhibition), the glucocorticosteroid budesonide (49% inhibition), the β _2_ adrenergic agent albuterol (71% inhibition), and the histamine H_1_-antagonist diphenhydramine (47% inhibition). The reduced response in the presence of either diphenhydramine or atropine suggests that both cholinergic (vagal) and H_1_-mediated pathways play a role in the toxin-induced bronchoconstriction. It is important to note that we previously showed that the dose of atropine used in these studies does not block histamine-induced bronchoconstriction (Abraham et al. 2005). These results are consistent with histamine stimulation of H_1_ receptors on nerves. The protective effects of albuterol and budesonide are also consistent with the involvement of mast cells/basophils because both agents have been reported to reduce mediator release (Wanner et al. 1987). Although albuterol can inhibit mediator release, its primary action is to relax smooth muscle. As illustrated in Figure 2, giving albuterol immediately after toxin challenge causes a rapid reversal of the response. The dual action of albuterol may account for its increased efficacy when compared with diphenhydramine (Figure 1).

**Figure 3:**
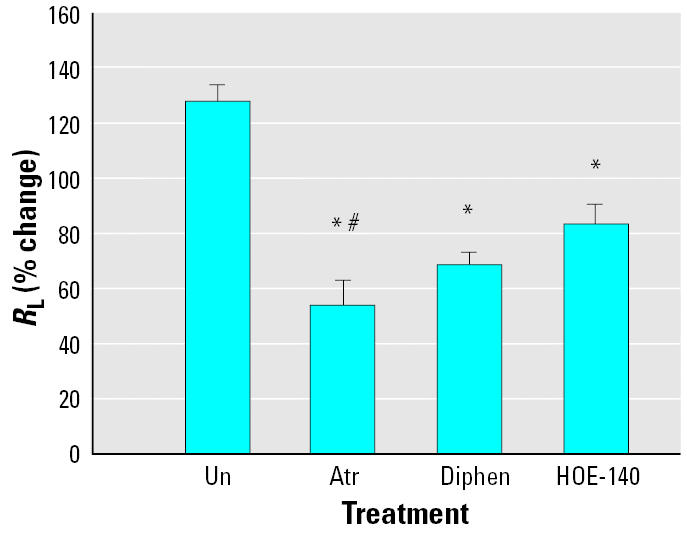
Effect of the bradykinin B_2_ receptor antagonist HOE-140 on crude brevetoxin–induced bronchoconstriction. Animals treated with HOE-140 had a reduced response compared with that seen when the animals were untreated (Un). The response in the presence of HOE-140 was similar to that seen with diphenhydramine (Diphen) but less than that seen with atropine (Atr). Values are mean ± SE for five sheep. 
*p < 0.05 versus untreated; ^#^*p* < 0.05 versus HOE-140.

Figure 3 illustrates that HOE–140, a bradykinin B_2_ receptor antagonist, significantly reduces the toxin-mediated bronchoconstriction. The 34% protection was significant when compared with the response when the animals were untreated but is significantly less than that seen with atropine (58%). These data indicate that, as with other types of irritants, brevetoxin increases kinin levels in the airways and that this inflammatory mediator contributes to toxin-induced airway responses.

### Cutaneous responses to toxin.

**Figure 4:**
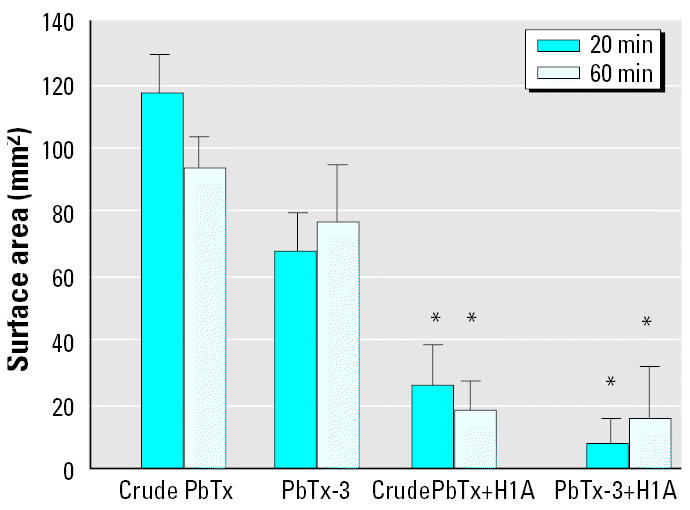
Effect of the histamine H_1_-antagonist diphenhydramine (H1A) on the ICRs to crude brevetoxins (PbTx) and PbTx-3. Values are mean ± SE for nine sheep. 
*p < 0.05 versus untreated.

The *in vivo* airway data support a role for a histamine H_1_-mediated component in the bronchoconstrictor response. To further investigate the potential for a generalized H_1_-mediated event, we performed skin tests with crude brevetoxins and pure PbTx-3. Both crude brevetoxins and PbTx-3 induced an ICR, which was reduced when the animals were pretreated with the H_1_-antagonist diphenhydramine (Figure 4). As expected, atropine had no effect on the ICR (data not shown). Thus, the collective data obtained with inhaled and injected toxins support the hypothesis that histamine H_1_-mediated pathways contribute to the *in vivo* effects of toxin.

### Allergen–toxin interactions.

**Figure 5:**
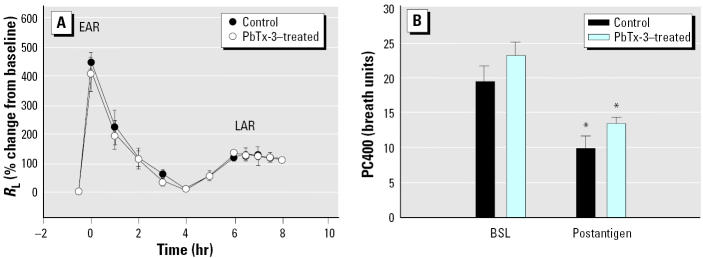
(*A*) The effect of 3 consecutive days of PbTx-3 exposure on antigen-induced EAR (early increase in *R*_L_, 0–4 hr) and LAR (late increase in *R*_L_, 4–8 hr; and (*B*) AHR to carbachol (indicated by the decreased PC400 24 hr after challenge). PbTx-3 exposure (20 breaths of 100 pg/mL) had no effect on any parameter. Values are mean ± SE for three sheep. 
*p < 0.05 versus baseline.

Our results indicate that inhaled toxin induces histamine and kinin release in the airways. Because these mediators are key components of allergen-induced airway responses, we wanted to determine if toxin challenge would induce a LAR and AHR similar to that seen with allergen. We also wanted to determine if preexposure to toxin could accentuate the response to allergen or if allergen challenge affected the response to toxin. We found that although PbTx-3 (20 breaths of 100 pg/mL) caused an early bronchoconstriction, there was no subsequent LAR or AHR in sheep that demonstrated these responses to inhaled allergen (data not shown). To determine if toxin exposure would enhance the response to allergen and/or affect the postantigen-induced AHR, we exposed three sheep to PbTx-3 (20 breaths of 100 pg/mL) for 3 consecutive days and then, on the day 4, measured the response to allergen challenge. On the next day, we measured their PC400 to carbachol to determine if the postantigen-induced AHR was affected. Figure 5 shows that the 3-day exposure to PbTx-3 did not alter the airway responses to allergen (EAR, LAR, or AHR) in these animals when compared with the control allergen responses in these same sheep.

**Figure 6:**
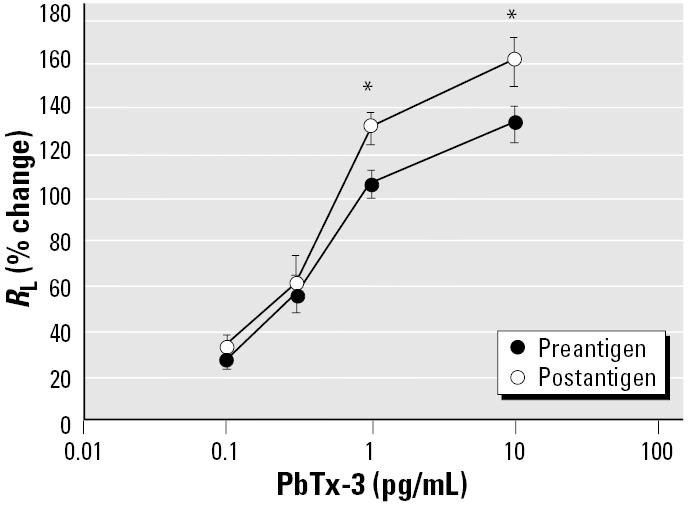
Effect of allergen challenge on the response to PbTx-3. Animals were more sensitive (indicated by the leftward shift in the concentration–response curve) to inhaled PbTx-3 one day after antigen challenge (postantigen) compared with before challenge (preantigen). The animals also demonstrated AHR to carbachol at this time, as indicated by a fall in the PC400, as described in the text. Values are mean ± SE for five sheep. 
*p < 0.05 versus preantigen.

Although preexposure to PbTx-3 did not accentuate antigen-induced responses, allergen challenge did increase the sensitivity to PbTx-3. Figure 6 shows that the concentration–response curve for PbTx-3 was significantly enhanced 24-hr after allergen exposure in sheep that develop a LAR (138 ± 9%). The sheep were also hyperresponsive to carbachol at this time as evidenced by the decrease in the post challenge PC400 (15 ± 1 breath units) when compared with the prechallenge PC400 (28 ± 2 breath units, *p* < 0.05). These data suggest that, as seen with other irritants, the airways are more sensitive to toxin after an asthma exacerbation.

## Discussion

The results of this study confirm and extend our previous work with brevetoxin aerosols (Abraham et al. 2005). We confirm that both cholinergic and histamine H_1_-mediated path-ways contribute to the bronchoconstrictor responses to toxin at concentrations 10–100 times greater than those used previously (Abraham et al. 2005). In addition, we identified standard asthma medications that protect against and/or reverse the constrictor effects of inhaled toxin. Our previous work showing that cromolyn sodium can block toxin-induced constrictor effects (Abraham et al. 2005) in conjunction with our current evidence that both the airway and cutaneous responses to toxin are histamine mediated, provides further support for the hypothesis that toxin activates mast cells *in vivo*. We show that kinins are released in the airways after toxin inhalation. This is a novel finding and, combined with the demonstration that the response to inhaled toxin is greater in inflamed airways, suggests that kinins could be contributing factors to the reported increased sensitivity of asthmatic patients to toxin exposure.

The methodology used in the airway provocation studies is designed to deliver toxin directly into the lung under controlled conditions. Although this technique does not mimic natural exposures because it bypasses the upper airways, it provides a reproducible controlled airway challenge system that eliminates dosing variability. This reproducibility is an important consideration when studying adverse lower airway effects and identifying protective pharmacologic agents. Given this, our findings should be applicable to humans experiencing lower airway symptoms during a red tide event.

Field studies indicate that humans with normal or healthy airways as well as those with compromised airways respond to a red tide event (Backer et al. 2003, 2005; Fleming et al. 2005; Kirkpatrick et al. 2004). Data from our previous study, using both allergic and nonallergic sheep, are consistent with these observations (Abraham et al. 2005). We found that the constrictor response to aerosolized brevetoxins was not limited to allergic animals. Normal (i.e., nonallergic) animals responded with a dose-dependent bronchoconstrictor response to inhaled toxin, and although there was tendency for the allergic animals to be more responsive, the difference was not significant. The similarity in responsiveness between the two groups was attributed to the fact that the allergic sheep had not seen allergen during the course of the studies and so the inflammatory status of their airways was similar to that of the nonallergic animals (Abraham et al. 2005). However, when the airways of allergic sheep are inflamed, as is the case after an antigen challenge (Abraham et al. 1994, 2000), the response to brevetoxin is enhanced (Figure 6). Thus, the response to toxin is not linked to the allergic status per se but is affected by the inflammatory status of the airways at the time of toxin exposure.

Even though toxin can elicit respiratory symptoms in both normal and compromised airways, a major aim of the present study was to identify asthma medications that could provide protective and/or therapeutic effects against toxin-induced bronchoconstriction. We used crude brevetoxins for these studies because we considered this aerosol more relevant to natural exposures experienced by persons living in coastal areas. To provide a more severe stimulus, higher concentrations of toxin were administered than used previously. The constrictor responses to PbTx-3 and crude brevetoxins at concentrations 10-to 100-fold lower were similar to those seen here and were inhibited by cromolyn sodium, atropine, and diphenhydramine (Abraham et al. 2005). Although atropine and diphenhydramine are not considered standard therapy for the treatment of asthma, the findings that these drugs blocked the responses to an increased toxin burden are important because they confirm that the protective effects seen previously were not dose dependent and that the same mechanisms are operative at higher toxin levels. Pretreatment with inhaled budesonide and albuterol provided significant protection against the toxin-induced bronchoconstriction. Acutely administered, the glucocorticosteroid budesonide is not a bronchodilator nor does it affect cholinergic responsiveness; therefore, its acute protective effects may be related to the drug’s ability to prevent mast cell activation *in vivo* (Denburg 1997; Kamada and Szefler 1995). This action would reduce the histaminergic component of the response. β _2_ adrenergic agents, in addition to their bronchodilatory activity, can also reduce mediator release (Wanner et al. 1987); therefore, such a mechanism could contribute to and possibly explain the slightly enhanced protection afforded by albuterol (Figure 1). The bronchodilatory activity normally attributed to β _2_ adrenergic agents is illustrated in Figure 2, where the albuterol is shown to reverse the toxin-induced bronchoconstriction. The latter effect is relevant to affected individuals that require rescue medication when exposed to aerosolized red tide toxins.

We previously reported that inhalation challenge with a variety of noxious agents results in increased tissue kallikrein activity and subsequent kinin generation in the airways. Increased kinin levels are associated with bronchoconstriction, inflammation, and AHR (Forteza et al. 1994, 1996; Scuri et al. 2000). Kinins also cause airway wall edema, which can influence the relaxation of the airways after a contractile stimulus (Wagner 1997). Asthmatic subjects are more sensitive to the effect of inhaled kinins than are normal individuals (Fuller et al. 1987). Inhaled bradykinin caused cough and retrosternal discomfort in both normal and asthmatic subjects, but caused bronchoconstriction only in the asthmatics (Fuller et al. 1987). Interestingly, this difference in kinin sensitivity could explain why Backer et al. 2003, 2005) were unable to demonstrate pulmonary function changes in normal subjects exposed to toxin even though they complained of chest discomfort, as opposed to Fleming et al. (2005), who were able to demonstrate pulmonary function changes in toxin-exposed asthmatics. Thus, kinin generation could be a contributing mechanism responsible for the heightened sensitivity of asthmatic subjects to toxin. Our data showing that the bradykinin B_2_ receptor antagonist HOE-140 significantly reduced the bronchoconstrictor response to inhaled toxin suggest that toxin stimulated increased kinin levels in the airway. These findings may be of greater consequence in terms of repeated exposures where increased kinins could contribute to inflammation and AHR (Zaias et al. 2004). Given the increased sensitivity of asthmatics to bradykinin (Fuller et al. 1987), our findings suggest that activation of the kinin pathway by brevetoxins could be another mechanism that contributes to more severe responses in patients with compromised airways.

In allergic sheep, the ICR to injected *A. suum* antigen is mast cell mediated, and antihistamines block this response (Ahmed et al. 1993; Lucio et al. 1992). We have used skin tests in combination with inhalation challenge tests to demonstrate that tryptase activates mast cells (Molinari et al. 1995, 1996). The data suggesting that inhaled toxin stimulates airway mast cells/basophils led us to test this hypothesis in the skin. Both crude brevetoxins and PbTx-3 induced ICRs, which were significantly reduced in the presence of diphenhydramine. These findings provide additional evidence that toxin activates mast cells *in vivo*. We should caution, however, that although the collective airway and skin data strongly argue in favor of mast cell hypothesis, final validation must be withheld until histamine measurements are obtained from lung and skin fluids. Nevertheless, if the findings in the airways and skin can be extrapolated to humans, then one might speculate that normal subjects, who would not generally have access to inhaled steroids and/or β _2_ adrenergics, might obtain relief from toxin-induced irritation with antihistamines.

The collective data from the skin and the airways prompted a number of questions regarding toxin-allergen interactions. These questions were precipitated by reports of adverse respiratory events hours after the original toxin exposure in susceptible individuals. In an attempt to mimic this scenario in the laboratory, we determined if a single toxin exposure could stimulate the development of a LAR and AHR, similar to that seen with allergen. We were unable to demonstrate this effect. Similarly, we were unable to show that exposures for 3 consecutive days to PbTx-3 accentuated the response to allergen. These findings indicate that, although toxin elicits an acute bronchoconstriction, the stimulus and/ or signaling mechanisms are not the same as seen with allergen that can result in a LAR and AHR. Furthermore, under the conditions of the experimental protocol, repeated toxin exposure was an insufficient stimulus to prime the airways such that the response to allergen challenge was augmented. We did find that allergen challenge accentuated the response to toxin. The increased responsiveness occurred during the period when the airways are actively inflamed and when they demonstrate increased responsiveness to other constricting agents, as seen previously (Abraham et al. 1994, 2000) and here with carbachol.

Although the data in the present study were generated in an acute setting, there is a parallel to the more chronic situation that exists in asthmatics whose airways are chronically inflamed and have heightened sensitivity to a variety of nonspecific bronchoconstrictors. Thus, the contention that the airways of asthmatics may be more sensitive to inhaled toxins than those of persons with normal airways may depend, in part, on the status of airway inflammation at the time of exposure.

In summary, we have provided new data concerning the airway effects of inhaled brevetoxins. We have identified clinical drugs that may be useful in the prevention and treatment of toxin-induced bronchial responses. Confirmation of these results must await controlled clinical trials. Nevertheless, given the caveat that these findings are based on experimental data collected in animals, our results may be of benefit to individuals affected by aerosolized brevetoxins at environmentally relevant levels.
